# 

**Published:** 1885-07

**Authors:** 


JULY, 1885.

NUMBER 12, VOLUME XXIV.

### THE BUFFALO Medical and Surgical Journal.

EDITORS:

THOS. LOTHROP, M. D. A. R. DAVIDSON, M. D.

PUBLISHED BY THE MEDICAL JOURNAL ASSOCIATION.

No. 8 WEST CHIPPEWA ST.

Entered at the Post-Office at Buffalo, N. Y., as Second-class Mail Matter.

